# p21-Activated Kinase 4 Signaling Promotes Japanese Encephalitis Virus-Mediated Inflammation in Astrocytes

**DOI:** 10.3389/fcimb.2017.00271

**Published:** 2017-06-21

**Authors:** Wen He, Zikai Zhao, Awais Anees, Yunchuan Li, Usama Ashraf, Zheng Chen, Yunfeng Song, Huanchun Chen, Shengbo Cao, Jing Ye

**Affiliations:** ^1^State Key Laboratory of Agricultural Microbiology, Huazhong Agricultural UniversityWuhan, China; ^2^Laboratory of Animal Virology, College of Veterinary Medicine, Huazhong Agricultural UniversityWuhan, China; ^3^The Cooperative Innovation Center for Sustainable Pig Production, Huazhong Agricultural UniversityWuhan, China; ^4^College of Life Science and Technology, Huazhong Agricultural UniversityWuhan, China

**Keywords:** JEV, PAK4, inflammation, MAPK, astrocyte

## Abstract

Japanese encephalitis virus (JEV) targets central nervous system, resulting in neuroinflammation with typical features of neuronal death along with hyper activation of glial cells. Exploring the mechanisms responsible for the JEV-caused inflammatory response remains a pivotal area of research. In the present study, we have explored the function of p21-activated kinase 4 (PAK4) in JEV-mediated inflammatory response in human astrocytes. The results showed that JEV infection enhances the phosphorylation of PAK4 in U251 cells and mouse brain. Knockdown of PAK4 resulted in decreased expression of inflammatory cytokines that include tumor necrosis factor alpha, interleukin-6, interleukin-1β, and chemokine (C-C motif) ligand 5 and interferon β upon JEV infection, suggesting that PAK4 signaling promotes JEV-mediated inflammation. In addition, we found that knockdown of PAK4 led to the inhibition of MAPK signaling including ERK, p38 MAPK and JNK, and also resulted in the reduced nuclear translocation of NF-κB and phosphorylation of AP-1. These results demonstrate that PAK4 signaling actively promotes JEV-mediated inflammation in human astrocytes via MAPK-NF-κB/AP-1 pathway, which will provide a new insight into the molecular mechanism of the JEV-induced inflammatory response.

## Introduction

Japanese encephalitis virus (JEV) is a mosquito-borne virus belonging to the *Flaviviridae* family of the viruses. JEV is highly prevalent cause of acute viral encephalitis in the Southeast Asian and the Western Pacific region of the world. Approximately, 35,000–50,000 Japanese encephalitis (JE) cases are recorded annually in the region with 10,000 deaths; nearly half of the survivors suffer from permanent neuropsychiatric sequelae (Vaughn and Hoke, 1992; Endy and Nisalak, 2002). Pathologically, infection of central nervous system (CNS) with JEV leads to severe disease, even fatal encephalitis (Solomon et al., 2000). Activation of glia (microglia and astrocytes), encroachment of inflammatory cells, rampant production of proinflammatory cytokines, and neuropathy are the idiosyncratic features of JE (German et al., 2006; Ghoshal et al., 2007).

Glial cells are the resident immune cells in the brain, and play crucial roles during neuroinflammation (Chen et al., 2000; Olson and Miller, 2004). It has been shown that JEV can infect astrocytes and microglia, and thus, these glial cells may serve as long-standing reservoir for JEV (Chen et al., 2000, 2004; Thongtan et al., 2010). The production of various proinflammatory mediators has been implicated in the process of activation of microglia and astrocytes following JEV infection (Chen et al., 2000; Bhowmick et al., 2007; Ghoshal et al., 2007). As the CNS professional macrophages, most of recent studies on inflammatory responses against JEV in the CNS have focused on microglia. However, increasing evidence suggests that astrocytes also play a critical role in the regulation of the cerebral inflammatory response (Bsibsi et al., 2002; Bowman et al., 2003; Liu et al., 2004; Carpentier et al., 2005; Park et al., 2006; Sterka et al., 2006). It has been reported that JEV differentially modulates the induction of multiple pro-inflammatory mediators in human astrocytoma and astroglioma cell-lines (Chen et al., 2010, 2011). However, the molecular mechanisms underlying the JEV-caused inflammatory response in astrocytes are largely unclear.

The p21-activated kinases (PAKs) are serine/threonine kinases that are indispensable effectors for RHO GTPases. They have been reported as well-known regulators of several oncogenic signaling cascades, nuclear signaling, cellular motility, and cytoskeletal organization (Kumar et al., 2006). Generally, PAKs are classified into two subgroups: subgroup I PAKs (PAK1–PAK3) and subgroup II PAKs (PAK4–PAK6). Of these, PAK4 is the member that has been studied extensively. Multiple types of tumors such as pulmonary, mammary, ovarian, and colorectal tumors, have been associated with increased expression level of PAK4 (Callow et al., 2002; Parsons et al., 2005; Chen et al., 2008; Kimmelman et al., 2008; Whale et al., 2011). Furthermore, upregulated pattern of PAK4 has been found to be linked with poor prognosis of ovarian tumor (Siu et al., 2010). Besides this, a functional cooperativity between PAK4 and MMP2 has also been established in governing resistance to anoikis-mediated cell death, invasion, and migration in glioma (Kesanakurti et al., 2012). Recent studies reveal that PAK4 is involved in ERK- and Akt-mediated augmentation of NF-κB signaling (Tyagi et al., 2014), which is well-known to be closely related to cell growth and inflammation. PAK4 inhibition by PF-3758309 suppresses NF-κB pathway and paves the way to downregulation of MMP- 2/MMP-9 expressions.

Given the close relationship of PAK4 function with glioma and NF-κB signaling, we wondered whether PAK4 also plays a role in neuroinflammation. In the present study, JEV infection was found to induce the activation of PAK4 in astrocytes, and its activation is associated with the increased expression of inflammatory cytokines mediated by JEV. Furthermore, PAK4 was shown to regulate mitogen-activated protein kinase (MAPK) and NF-κB/AP-1 signaling pathway during JEV infection. These results delivered first experimental evidence for involvement of PAK4 in JEV-caused inflammatory response in astrocytes.

## Materials and methods

### Cell culture, virus propagation and viral infection

Human glioma cell line U251 (also known as human glioblastoma or astrocytoma cell line) was cultured and maintained in Dulbecco's modified Eagle's medium (DMEM; 4,500 mg/liter glucose) supplemented with 10% (v/v) heat-inactivated fetal bovine serum, penicillin (100 U/ml), and streptomycin sulfate (100 mg/ml) at 37°C in a 5% CO2 atmosphere. JEV wild-type strain P3 was propagated in suckling mouse brains (the animal experiments were performed in accordance with the National Institutes of Health's Guide for the Care and Use of Laboratory Animals, and the experimental protocols were approved by the Huazhong Agricultural University's Research Ethics Committee of the College of Veterinary Medicine), and viral titer was determined by plaque assay on BHK-21 cells. U251 cells were plated in six-well plates (6 × 10^5^ cells/well) and grown to 80% confluence. Cells were subsequently infected with JEV P3 strain at multiplicity of infection (MOI) of 5.

### Reagents

Antibodies against PAK4, GAPDH, p65, and Lamin A, ERK1/2, phosphor-ERK1/2, P38MAPK, phosphor-P38MAPK, JNK1, phosphor-JNK1, c-JUN phosphor-c-JUN, and IκBα were purchased from Abclonal Technology (Wuhan, China). Antibody against phosphor-PAK4 was obtained from Cell Signaling Technology (Beverly, MA). HRP-labeled anti-mouse/rabbit secondary Antibodies (Boster, China) were used in this study. The monoclonal antibody against JEV NS5 was prepared in our laboratory.

### JEV infection in mice

Adult BALB/c mice (8 week old) were purchased from the Hubei Provincial Center for Disease Control and Prevention (Wuhan, China). For PAK4 detection in mouse brain, mice were injected i.p. with 10^6^ PFU JEV P3 strain in 200 ml PBS. The remaining mice were sacrificed on day 6 post infection, and brain samples were collected.

### RNA extraction, cDNA synthesis, and quantitative real-time PCR (qRT-PCR) analysis

Cellular RNA was isolated with TRIzol reagent (Invitrogen), and subsequently, was reverse transcribed into cDNA using commercially available First Strand cDNA Synthesis Kit (TOYOBO) following the manufacturer's instructions. The qRT-PCR analysis was done by using SYBR Green PCR Master Mix (TOYOBO) and a 7500 Real-time PCR System (Applied Biosystems). Results were normalized to β-actin expression in each sample. Primers sequences were as follows: human β-actin 5′- AGCGGGAAATCGTGCGTGAC-3′ (sense) and 5′-GGAAGGAAGGCTGGAAGAGTG-3′ (antisense); human CCL5 5′- GTGGCA ATGAGGATGACTTGT-3′ (sense) and 5′- AGATGAAGGGAAAGAAGGTGCT-3′ (antisense); human IL-1β 5′-CCAGGAGAAGATTCCAAAGATG-3′ (sense) and 5′- AGGAACTGGATCAGGACTTTTG-3′ (antisense); human IL-6 5′-CTGTCATCCTCATTGCTACTGC-3′ (sense) and 5′-ATGTACTCCCGAACCCATTTCT-3′ (antisense); human TNF-α 5′-TGTAGCCCATGTTGTAGCAAAC-3′ (sense) and 5′- ACTCGGCAAAGTCGAGATAGTC-3′ (antisense).

### Immunofluorescence assay

After attaining 80% growth confluency, the cells were washed with DMEM before virus inoculation. After washing, the cells were either mock-infected or JEV-infected at an MOI of 1, 2, or 5 for 1 h. The supernatant was removed and the cells were incubated with 3% serum maintenance solution. At 36 h after virus infection, the cells were blocked with 1% BSA in PBS (pH = 7.2) for 30 min. Then, cells were stained with the monoclonal antibody specific to JEV NS5 for a period of 1 h. After washing for three times with PBS, the cells were incubated with Alexa Fluor 488-labeled secondary antibody (Invitrogen) for 30 min and then the nuclei were stained with DAPI (Invitrogen). After the staining, cells were observed using a fluorescence microscope (Zeiss) at 20× magnification.

### Plasmid construction

To construct the plasmid encoding PAK4, the coding region of PAK4 gene were amplified from cDNA derived from HeLa cells by PCR and cloned into pcDNA3.1 to yield pPAK4.

### RNA interference

Small interfering RNAs (siRNAs) against human PAK4 (5′- UUCUGCUCGUGCUGGUCGAAGTT-3′), TLR3 (5′-CGAAUUUGACUGAACUCCA-3′), and RIG-I (5′-GAGGUGCAGUAUAUUCAGG-3′) and the negative control siRNA were purchased from Gene Pharma. Cells were transfected with 50 nM of each siRNA by using Lipofectamine2000.

### Western blotting

Total cellular lysates were obtained by lysing cells in radioimmunoprecipitation assay buffer (RIPA buffer) containing protease and phosphatase inhibitors (Roche, Mannheim, Germany). The concentration of proteins was determined using BCA Protein Assay Kit (Thermo Scientific). SDS-PAGE was performed, and then proteins were blotted on nitrocellulose membrane. The membrane was blocked with a blocking solution (TBST+5%BSA) for 1 h and incubated with the primary antibody for 2 h. After washing for three times, the membrane was immersed with appropriate peroxidase-conjugated secondary antibodies (Boster). Detection of proteins was conducted by using ECL reagent (Thermo Scientific).

## Results

### JEV-infection induces the phosphorylation of PAK4 in human astrocytes

**Figure 1:**
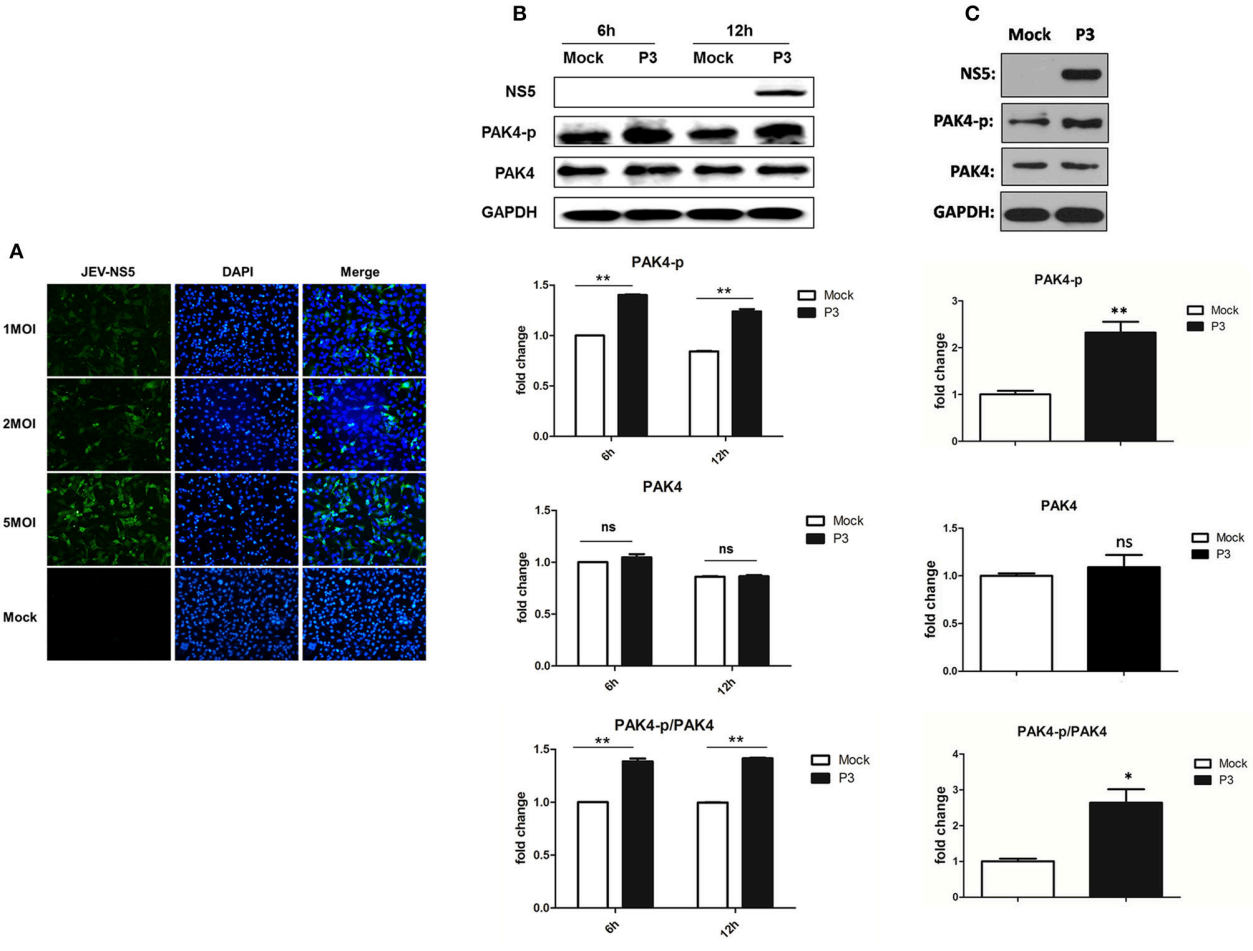
JEV infection promotes PAK4 phosphorylation in U251. **(A)** U251 cells were mock-infected or infected with JEV P3 strain at an MOI of 1, 2, or 5. At 36 h after virus infection, JEV NS5 was detected by immunofluorescence assay. The nuclei were stained with DAPI. After the staining, cells were observed using a fluorescence microscope (Zeiss) at 20 × magnification. **(B)** U251 cells were mock-infected or infected with JEV P3 strain at MOI of 5. Cells were harvested at 6 and 12 h post infection and the expression and phosphorylation of PAK4 were detected by using Western-blot. **(C)** Mice were infected with JEV or mock infected with PBS, and brain samples were collected after 6 days for analysis of phosphor-PAK4 and PAK4 expression using Western-blot. Protein and phosphorylation levels of PAK4 were quantified with immunoblot scanning and normalized to the amount of GAPDH. ^*^p < 0.05, ^**^p < 0.01, ns: no significance, compared with mock-infected cells (*n* = 3).

To examine the infectivity of JEV on astrocytes, U251 cells were mock-infected or infected with 1, 2, or 5 MOI of JEV. Cells were fixed at 24 h post infection and the expression of JEV NS5 protein was detected by immunofluorescence assay. We found that U251 cells were successfully infected by JEV and the infection rate was presented in a dose-dependent manner (Figure 1A). To investigate the role of PAK4 in JEV infected astrocytes, cells were mock-infected or JEV-infected at an MOI of 5. Subsequently, cells were harvested at 6 and 12 h post infection and the expression and phosphorylation of PAK4 were detected by using Western-blot. We observed higher level of phosphorylated PAK4 in JEV-infected cells as compared to uninfected-cells at both time points (Figure 1B). However, no significant change was shown on expression level of PAK4 after JEV infection. These results suggest that JEV-infection can induce the activation of PAK4 in astrocytes. To verify this result *in vivo*, phosphorylation of PAK4 was detected in brain tissue of JEV-infected mice. As expected, phosphorylated-PAK4 was significantly upregulated upon JEV infection in mouse brain (Figure 1C).

### Activation of PAK4 promotes the inflammatory response induced by JEV

**Figure 2:**
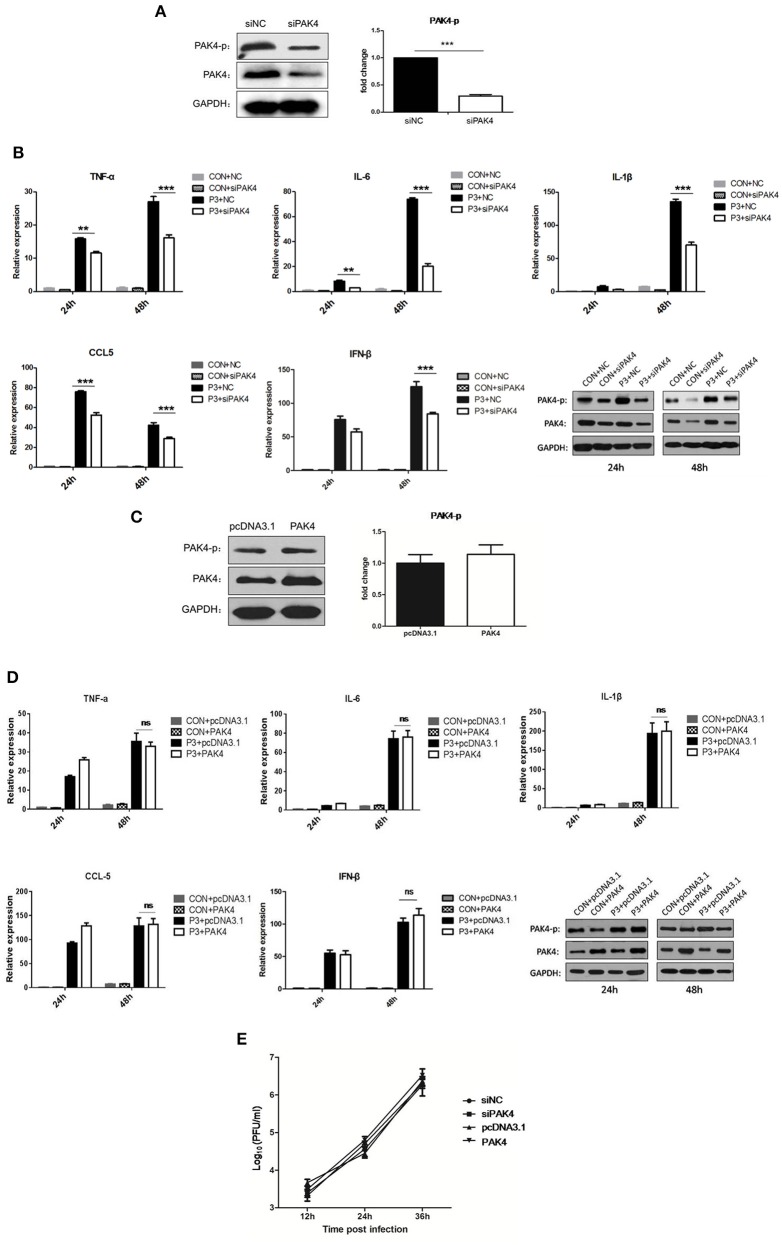
PAK4 signaling contributes to JEV-induced inflammation in U251 cells. **(A)** U251 cells were transfected with siRNA targeting PAK4 (siPAK4) or negative control siRNA (siNC). Cells were collected at 36 h post transfection and the PAK4 was detected by Western-blot. Levels of PAK4 were quantified with immunoblot scanning and normalized to the amount of GAPDH. ^***^p < 0.001, compared with cells transfected with siNC (*n* = 3). **(B)** U251 cells were transfected with siPAK4 or siNC, followed by JEV infection. Cells were harvested at 24 and 48 h post infection and mRNA levels of inflammatory cytokines and IFN-β were determined by qRT-PCR. ^***^p < 0.001, ^**^p < 0.01, compared with cells transfected with siNC (*n* = 3). Protein levels of phospho-PAK4 and PAK4 were determined by Western-blot (downright panel). **(C)** U251 cells were transfected with plasmid encoding PAK4 (pPAK4) or empty vector pcDNA3.1. Cells were collected at 36 h post transfection and PAK4 was detected by Western-blot. Levels of PAK4 were quantified with immunoblot scanning and normalized to the amount of GAPDH (ns: no significant change). **(D)** U251 cells were transfected with plasmid encoding PAK4 or pcDNA3.1, followed by JEV-infection. Cells were harvested at 24 and 48 h post infection and mRNA levels of inflammatory cytokines and IFN-β were determined by qRT-PCR. Protein levels of phospho-PAK4 and PAK4 were determined by Western-blot (downright panel). **(E)** U251 cells were transfected with siPAK4, siNC, pPAK4, or pcDNA3.1, followed by JEV infection. Cell supernatants were harvested at 12, 24, and 36 h post infection and viral titers were determined by plaque assay on BHK-21.

To evaluate the role of PAK4 in JEV-induced inflammation in astrocytes, PAK4 specific siRNA (siPAK4) was employed. The cells were either transfected with siPAK4 or negative control siRNA (siNC) and the phosphorylation and expression of PAK4 was examined at 36 h post transfection. A significant reduction of PAK4 and phosphorylated PAK4 was shown in siPAK4-transfected cells (Figure 2A). Furthermore, cells were infected by JEV following transfection of siPAK4 or siNC. The mRNA levels of pro-inflammatory cytokines, including tumor necrosis factor alpha (TNF-α), interleukin-6 (IL-6), interleukin-1β (IL-1β), and chemokine (C-C motif) ligand 5 (CCL5), and type I interferon (IFN-β) were detected. We found that JEV infection significantly induced the expression of inflammatory cytokines and IFN-β, whereas knockdown of PAK4 remarkably reduced the levels of these cytokines elicited by JEV (Figure 2B), suggesting that PAK4 positively regulated the inflammatory response induced by JEV in astrocytes. To further confirm this result, plasmid encoding PAK4 gene was constructed and transfected into U251 cells. PAK4 was overexpressed in the cells, whereas no alteration of PAK4 phosphorylation was observed (Figure 2C). We subsequently examined the expression of pro-inflammatory cytokines and IFN-β in PAK4 overexpressed cells after JEV infection. No significant change of cytokine mRNA levels was shown upon PAK4 overexpression (Figure 2D), indicating that phosphorylation of PAK4 is required for promoting the inflammation response in JEV-infected cells. To determine whether the activation of PAK4 affects JEV replication, we monitored the viral titers in siRNA and plasmid-transfected cells. Viral replication was unaffected by knockdown or overexpression of PAK4, indicating that targeting the inflammatory response may not prevent JEV replication in U251 cells (Figure 2E).

### PAK4 is involved in the activation of MAPK signaling in JEV infected astrocytes

**Figure 3:**
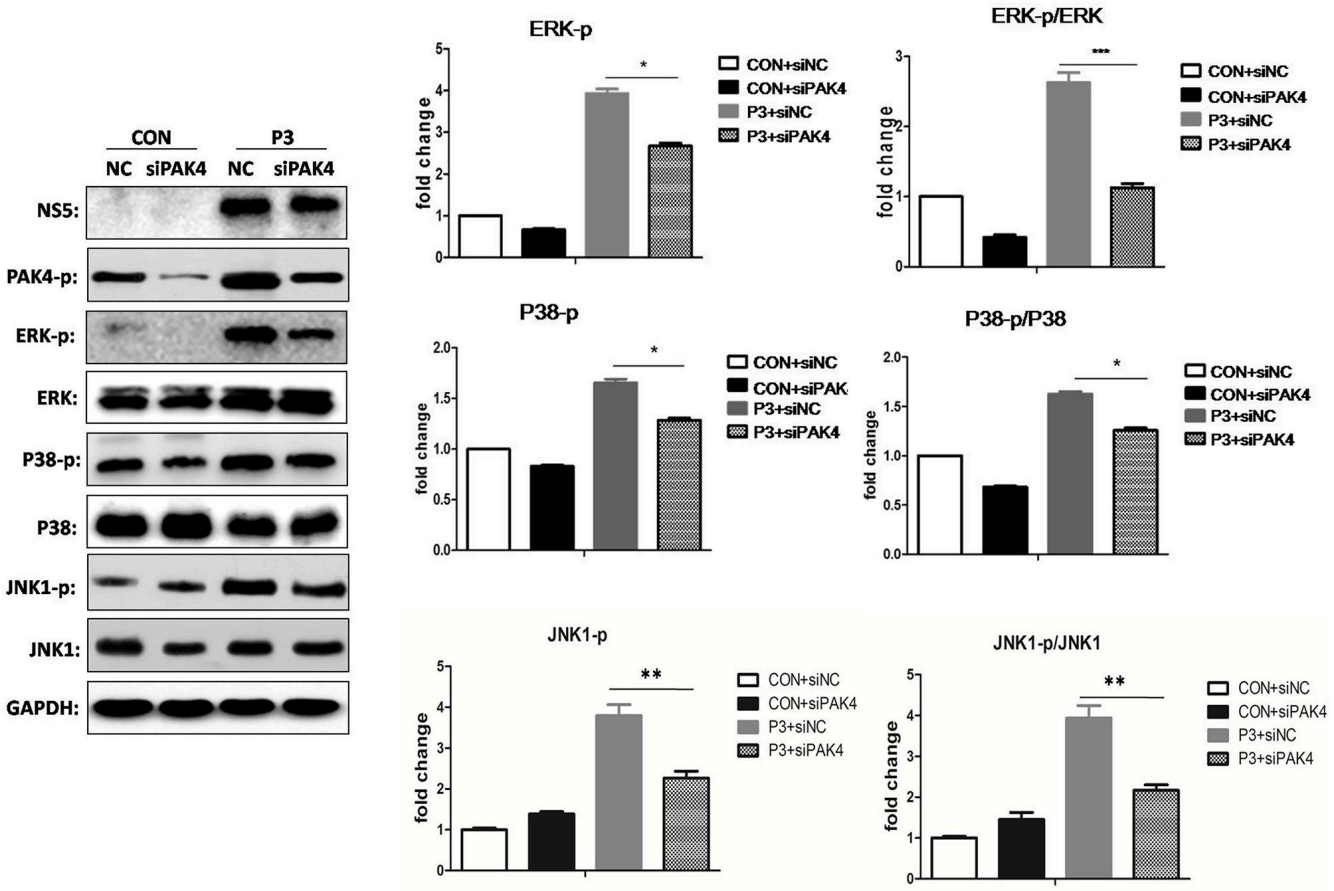
PAK4 positively regulates MAPK signaling during JEV infection. U251 cells were transfected with siPAK4 or siNC, followed by JEV infection. Cells were harvested at 12 h post infection and expression and phosphorylation of ERK1/2, P38 MAPK and JNK1 were detected by Western-blot. Levels of phosphorylated ERK, P38MAPK and JNK1, and the ratio of p-ERK, p-P38MAPK and p-JNK1 were quantified with immunoblot scanning and normalized to the amount of GAPDH. ^***^p < 0.001, ^**^p < 0.01, ^*^p < 0.05, compared with cells transfected with siNC (*n* = 3).

As well-known that PAK4 is involved in the activation of ERK signaling; we further investigated the role of PAK4 on ERK activation during JEV infection. U251 cells were transfected with siPAK4 or siNC followed by infection of JEV and ERK phosphorylation and expression were detected by Western-blot. As expected, JEV infection increased the phosphorylation level of ERK1/2, while a reduction of phosphor-ERK1/2 was observed in cells transfected with siPAK4 (Figure 3), suggesting PAK4 positively regulates ERK activation. To further characterize the regulation role of PAK4 on other members of MAPK family during JEV infection, phosphorylation of p38MAPK and JNK1 was also detected. Our results revealed that knockdown of PAK4 significantly inhibited the phosphorylation of p38MAPK and JNK1 during JEV-infection (Figure 3). These results demonstrate that PAK4 contributes to the activation of MAPK signaling during JEV infection.

### PAK4 signaling contributes to the activation of NF-κB and AP-1 in JEV infected astrocytes

**Figure 4:**
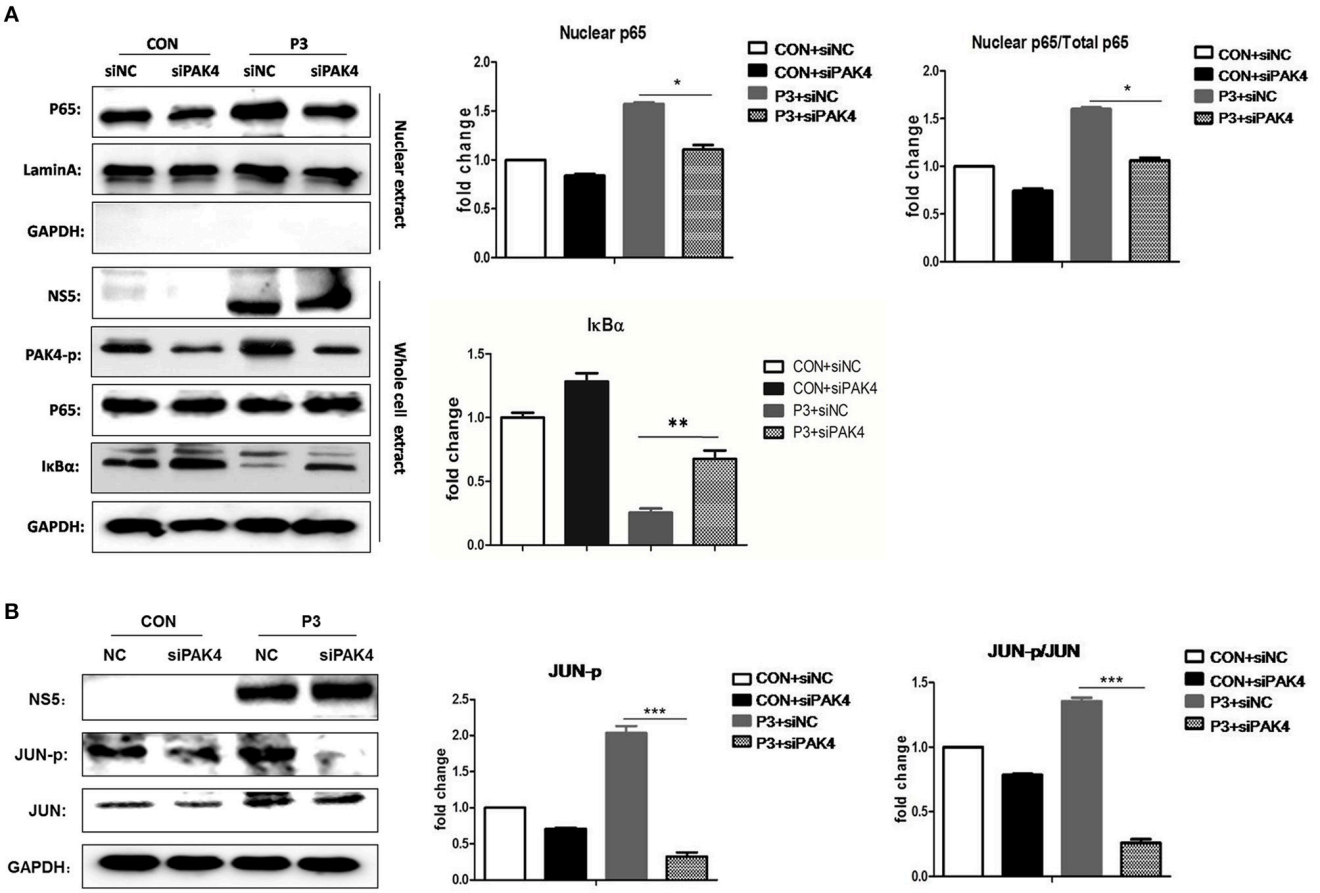
PAK4 promotes the activation of NF-κB and AP-1 in JEV-infected U251 cells. U251 cells were transfected with siPAK4 or siNC followed by JEV infection and were harvested at 12 h post infection. The nuclear translocation of p65, protein level of IκBα **(A)** and expression and phosphorylation of JUN **(B)** were detected by Western-blot. Levels of nuclear localized p65, IκBα and phosphorylated JUN were quantified with immunoblot scanning and normalized to the amount of GAPDH and Lamin A. ^***^p < 0.001, ^**^p < 0.01, ^*^p < 0.05, compared with cells transfected with siNC (*n* = 3).

It is well established that JEV-infection can induce the activation of NF-κB and AP-1 that are considered as key transcriptional factors of proinflammatory cytokines. To better understand the mechanism by which PAK4 regulates the inflammatory response in astrocytes, the impact of PAK4 on NF-κB and AP-1 activity in JEV-infected U251 cell line was subsequently investigated. U251 cells were transfected with siPAK4 or siNC followed by infection of JEV and nuclear translocation of p65, one of NF-κB subunits, and the degradation of IκBα, an upstream inhibitor of NF-κB, were detected by Western-blot. JEV-infection strongly induced the degradation of IκBα and enhanced the translocation of p65 from cytoplasm to the nucleus (Figure 4A). In contrast, the transfection of cells with siPAK4 significantly restored the level of IκBα and inhibited the nuclear translocation of p65 in JEV-infected U251. Subsequently, phosphorylation of c-Jun, an AP-1 subunit, were also detected by Western-blot. Similarly, the phosphorylation of c-Jun was obviously increased upon JEV infection, while siPAK4 transfection markedly attenuated the signal of AP-1 activation (Figure 4B). These findings suggest that PAK4 signaling could stimulate the activation of c and AP-1 in JEV infected astrocytes.

### JEV-induced PAK4 activation is dependent on TLR3 and RIG-I signaling

**Figure 5:**
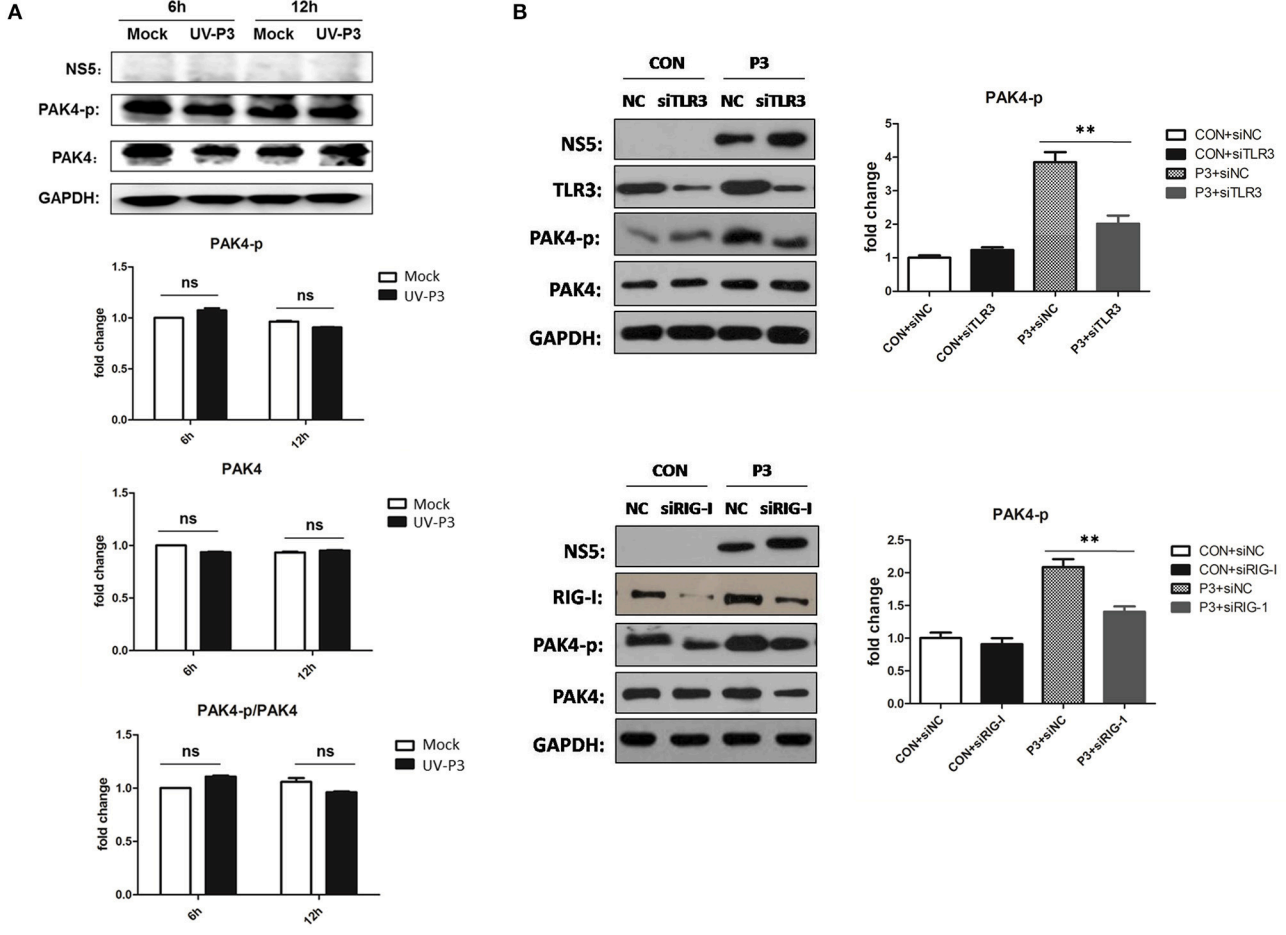
TLR3 and RIG-I signaling is involved in JEV-induced activation of PAK4 in astrocytes. **(A)** JEV P3 strain was irradiated with ultraviolet (UV) rays for 3 h. U251 cells were mock-infected or infected with UV-inactivated JEV P3 strain at MOI of 5. Cells were harvested at 6 and 12 h post infection and the expression and phosphorylation of PAK4 were detected by using Western-blot. Protein and phosphorylation levels of PAK4 were quantified with immunoblot scanning and normalized to the amount of GAPDH. **(B)** U251 cells were transfected with siRNA for TLR3 or RIG-I, or siNC, and infected with JEV at 24 h post transfection. After infection for 12 h, cells were collected and the phosphor-PAK4 were detected by Western-blot, and levels were quantified by immunoblot scanning and normalized to the amount of GAPDH. ^**^p < 0.01, compared with cells transfected with siNC (*n* = 3).

To further explore the mechanism for enhancing PAK4 activation by JEV infection, cells inoculated with UV-irradiated inactivated JEV were used for PAK4 detection. The phosophorylation and expression level of PAK4 were shown to have no significant changes (Figure 5A), indicating that productive infection of JEV is essential requirement for PAK4 activation. As it has been known that TLR3 and RIG-I signaling can mediate the expression of type I interferon and pro-inflammatory cytokines through recognizing double-strand RNA produced during JEV genomic replication, we next investigated the role of TLR3 and RIG-I on PAK4 activation in JEV-infected astrocytes. The results showed that levels of PAK4 phosphorylation enhanced by JEV infection were significantly reduced upon TLR3 or RIG-I knockdown in U251 cells (Figure 5B), indicating that JEV-induced PAK4 activation is dependent on TLR3 and RIG-I signaling.

## Discussion

JEV is an important neurotropic pathogen; causing severe neurological sequelae in humans. It induces neuroinflammation with features of viral encephalitis, including immune cell infiltration and neuronal degeneration (Misra and Kalita, 2010). The production of cytokines and chemokines from periphery or by glia is the main source of establishment of pro-inflammatory environment in the JEV-infected brain (Ghoshal et al., 2007). Although, microglia have been known as the main resident immune cells in the CNS and represent critical effectors of CNS inflammation (Aloisi, 2001; Olson and Miller, 2004), evidence is emerging that astrocytes also participate in releasing inflammatory cytokines and in enhancing localized inflammatory response (Correale and Farez, 2015; Rannikko et al., 2015; Ashraf et al., 2016). It has been demonstrated that JEV can replicate in astrocytes, and in turn, contribute to neuroinflammation (Chen et al., 2000; Bhowmick et al., 2007). However, the basic mechanisms how JEV triggers the astrocyte-mediated inflammatory response remain to be elucidated. Herein, we found that JEV infection promotes the activation of PAK4 in human astrocytes. We also demonstrated that PAK4 positively regulates MAPK-NF-κB/AP-1 signaling pathway and inflammatory response in JEV-infected astrocytes.

Kinase is a group of important signal molecules involved in different cellular pathways. Multiple kinase pathways, including AKT, MAPK, GSK and PKA, have been known to participate in the inflammation mediated by neuroinvasive viruses, such as JEV and WNV (Zhang et al., 2015; Ye et al., 2016). PAK4 belonging to subgroup II of PAKs, is highly expressed in a variety of cancers (Chen et al., 2008; Ahn et al., 2011; Mak et al., 2011; Cai et al., 2015). Many studies have demonstrated the roles of PAK4 in regulating tumor cell mobility, invasion, and proliferation (Paliouras et al., 2009; Kesanakurti et al., 2012; Ryu et al., 2014). Because of importance of PAK4 in triggering oncogenic signaling cascades and in cellular transformation, targeting the PAK4 therapeutically could be of valuable interest (Crawford et al., 2012). Although, the involvement of PAK4 in cancer development has been well established, few studies have implicated the role of PAK4 in inflammation. In this study, we have demonstrated a pathobiological role of PAK4 in inflammatory response of human astrocytes for the first time. Elevated levels of PAK4 have been examined in multiple types of cancer that include colorectal, ovarian, mammary, and gastric cancers. However, only an increased level of phosphor-PAK4, but not the expression of PAK4, was observed upon JEV-infection, suggesting that the upstream signaling regulating PAK4 activation in JEV-infected astrocytes may be different from other tumor cells. We also demonstrated a downregulation of type I interferon upon PAK4 inhibition during JEV-infection. However, viral replication was not affected by PAK4 knockdown. Similar phenomenon was also observed in our previous study which found that inhibition of RIG-I signaling reduced the IFN-β production whereas didn't limit the JEV replication (Zhu et al., 2015) and some other studies (Thounaojam et al., 2014a,b). This may be explained by the low fold change on IFN-β expression that not enough to exert effects on JEV replication and the strong inhibitory ability of JEV on type I interferon signaling (Lin et al., 2006).

Based on the studies conducted so far, it is suggested that PAK4 can activate several signaling pathways responsible for tumorigenesis (Kumar et al., 2006; Park et al., 2013; Tabusa et al., 2013; Radu et al., 2014). Recent studies also reveal that Akt and ERK played an important role in mediating the effect of PAK4 on subcellular localization of NF-κB/p65 and its transcriptional activity. This is in consistence with our previous finding which demonstrates the involvement of ERK pathways in the activation of NF-κB/p65 and the downstream inflammatory response in JEV-infected glial cells (Jiang et al., 2014). Here we confirmed that JEV-induced phosphorylation of PAK4 can mediate the activation of ERK-NF-κB pathway in astrocytes. Additionally, the activation of AP-1, another transcriptional factor responsible for the expression of inflammatory cytokines, was also shown to be regulated by PAK4 during JEV infection. These findings may be one of the mechanisms by which PAK4 positively regulates the expression of inflammatory cytokines in JEV-infected astrocytes. Except for ERKs, p38MAPK, and JNK also belong to MAPK family. The p38MAPK and JNK pathways are involved in controlling many different cellular processes such as cell differentiation, proliferation, and apoptosis. The roles of p38 and JNK signaling in stress response and inflammation have been well established by the research over the past two decades, and the role of JNK in JEV-induced neuroinflammation has also been determined in our previous research (Ye et al., 2016). Here our present study demonstrated p38MAPK and JNK1 as the novel downstream mediators of PAK4, suggesting p38MAPK and JNK pathways may also be involved in PAK4-regulated inflammation during JEV infection. These findings provide new evidence to support the important role of PAK4 in regulating MAPK signaling pathways. However, the molecular mechanisms engaged in PAK4-associated regulation of MAPK pathways are required to be explored in future studies.

Given that productive infection of JEV is required to stimulate PAK4 activation, we speculated that replication of viral genomic RNA may play a key role. As expected, our results confirmed that TLR3 and RIG-I signaling contribute to JEV-induced activation of PAK4. This is supported by the well-known role of TLR3 and RIG-I signaling in JEV-induced innate immunity. However, besides viral RNA, whether any other viral component(s), such as non-structural proteins, contribute to the PAK4 activation still needs further clarifications.

In summary, our results demonstrated for the first time that JEV infection stimulates the phosphorylation of PAK4 which in turn promotes JEV-induced inflammatory response in human astrocytes via triggering MAPK-NF-κB/AP-1 signaling cascades. These findings suggest that PAK4 could serve as a potential target for JE therapy.

## Author contributions

Conceived and designed the experiments: JY, SC, and HC. Performed the experiments: WH, ZZ, YL, and ZC. Analyzed the data: WH, JY, and YS. Wrote the paper: JY, AA, WH, and UA.

### Conflict of interest statement

The authors declare that the research was conducted in the absence of any commercial or financial relationships that could be construed as a potential conflict of interest.
